# Treatment of Orchitis

**Published:** 1876-08

**Authors:** 


Treatment of Orchitis.—In the *Lancet,* Dr. J. R. Beck details his treatment of orchitis as follows:

I premise with the declaration that while the application of the principle may have been original with me, yet the principle itself is a very old one, and therefore no credit attaches to my application of it. The point to be gained in the treatment, as it appears to me, is to reduce the swelling as rapidly as possible. This may be attained in numerous ways, notably by the old method of "strapping." But strapping the organ is open to two very serious objections: 1st, that it does not furnish an equable support and pressure; and 2d, that it has to be frequently renewed, and thus makes too much work for the surgeon. All this I avoid by simply taking what are called "male safes," or "condoms," two of which I combine by introducing the one within the other, and drawing this "glove," as it were, over the testicle. I have lately used the toy balloons, which are made of India-rubber for the same purpose. This is all I do for these cases. The rubber exercises equable and continued pressure upon the testicle, and the patient walks away very much happier than any punctured cases.—*Medical Brief.*

